# Role of Ion Channel Remodeling in Endothelial Dysfunction Induced by Pulmonary Arterial Hypertension

**DOI:** 10.3390/biom12040484

**Published:** 2022-03-22

**Authors:** Joana Santos-Gomes, Hélène Le Ribeuz, Carmen Brás-Silva, Fabrice Antigny, Rui Adão

**Affiliations:** 1 UnIC@RISE, Department of Surgery and Physiology, Faculty of Medicine, University of Porto, 4200-319 Porto, Portugal; joanapxgomes@gmail.com (J.S.-G.); carmensb@med.up.pt (C.B.-S.); 2 Faculté de Médecine, School of Medicine, Université Paris-Saclay, 94276 Le Kremlin-Bicêtre, France; helene.leribeuz@gmail.com (H.L.R.); antignyfabrice@gmail.com (F.A.); 3 INSERM UMR_S 999 Pulmonary Hypertension: Pathophysiology and Novel Therapies, Groupe Hospitalier Paris Saint-Joseph, Hôpital Marie Lannelongue, 92350 Le Plessis-Robinson, France; 4 Faculty of Nutrition and Food Sciences, University of Porto, Rua do Campo Alegre, 823, 4150-180 Porto, Portugal

**Keywords:** endothelium, voltage-gated ion channels, pulmonary hypertension, vascular pathology

## Abstract

Endothelial dysfunction is a key player in advancing vascular pathology in pulmonary arterial hypertension (PAH), a disease essentially characterized by intense remodeling of the pulmonary vasculature, vasoconstriction, endothelial dysfunction, inflammation, oxidative stress, and thrombosis in situ. These vascular features culminate in an increase in pulmonary vascular resistance, subsequent right heart failure, and premature death. Over the past years, there has been a great development in our understanding of pulmonary endothelial biology related to the genetic and molecular mechanisms that modulate the endothelial response to direct or indirect injury and how their dysregulation can promote PAH pathogenesis. Ion channels are key regulators of vasoconstriction and proliferative/apoptotic phenotypes; however, they are poorly studied at the endothelial level. The current review will describe and categorize different expression, functions, regulation, and remodeling of endothelial ion channels (K^+^, Ca^2+^, Na^+^, and Cl^−^ channels) in PAH. We will focus on the potential pathogenic role of ion channel deregulation in the onset and progression of endothelial dysfunction during the development of PAH and its potential therapeutic role.

## 1. Introduction

Endothelial cells (ECs) form a multifunctional signal-transducing surface that performs diverse tasks depending on its localization in the vessel tree. A diverse variety of ion channels is expressed in the plasma and organelle membranes that control the function of these cells [1]. For example, these channels can directly or indirectly regulate changes in intracellular Ca^2+^ concentration ([Ca^2+^]_i_) that serves as an essential second messenger monitoring the activity of Ca^2+^-dependent ion channels, cell membrane potential, production and release of many vasoactive factors, and regulating barrier function and proliferation of ECs [1].

Pulmonary arterial hypertension (PAH) is a severe disease characterized by vascular remodeling in pulmonary arteries attributable to persistent vasoconstriction, proliferation, inflammation, and in situ thrombosis. The pathogenesis of PAH involves a multifactorial process, and endothelial dysfunction seems to play an integral role in mediating the structural changes in the pulmonary vasculature [2]. Pulmonary arterial tone regulation may be due to variations in channel function, numbers of channels *per* cell, channel conductance, or open probability. The early variations in vascular remodeling include endothelial dysfunction, which is correlated to nitric oxide (NO) production and delivery [3,4]. The interactive link between smooth muscle cells (SMCs) and ECs is vital for controlling blood pressure and blood flow on a moment-to-moment basis [3]. Therefore, in PAH, the disease is directly associated with ion channels in ECs and SMCs.

Despite availability of effective current therapies, no cure exists for PAH, and lung transplantation remains the last therapy possibility for suitable PAH patients, with a 29% survival rate at 10 years post-transplantation [2]. Consequently, it is essential to clarify the distribution and mechanism of ions and their corresponding ion channels in PAH to potentially develop more effective drugs for the treatment of PAH [5]. Indeed, the identification of heterozygous loss-of-function mutations in the *KCNK3* (potassium two pore domain channel subfamily K member 3) gene that encodes the tandem of P domains in weak inward rectifier K^+^ channels (TWIK)-related acid-sensitive potassium channel 1 (TASK1) and loss-of-function mutation in the *ABCC8* (coding for adenosine triphosphate (ATP)-sensitive potassium channel subunit) as a cause for PAH, has revived interest in the concept of channelopathy [6]. Although ion channels are key regulators of vasoconstriction and proliferative/apoptotic phenotypes, they are poorly studied at the endothelial level.

**Figure 1:**
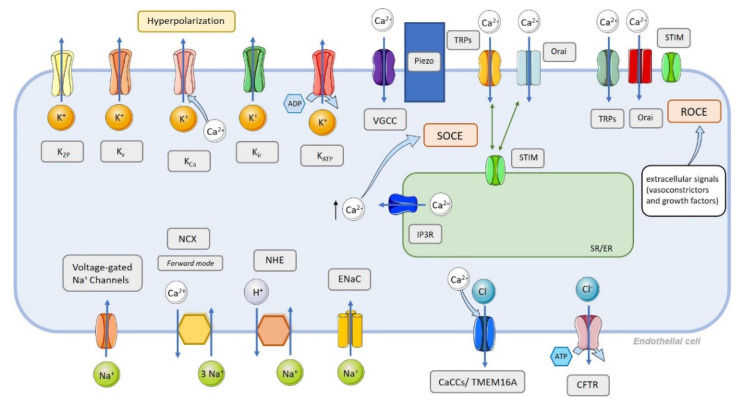
Overview of ion channels present in cells. ATP-sensitive K^+^ channel (K_ATP_); Ca^2+^-activated Cl^−^ channels (CaCCs); Ca^2+^-activated K^+^ channels (K_Ca_); cystic fibrosis transmembrane conductance regulator (CFTR); endoplasmic reticulum (ER); epithelial Na^+^ channels (ENaC); inositol triphosphate (IP3); inositol triphosphate receptor (IP3R); interacting molecule (STIM); inward rectifier K^+^ channels (K_ir_); Na^+^-H^+^ exchanger (NHE); Na^+^/Ca^2+^ exchanger (NCX); receptor-operated Ca^2+^ entry (ROCE); receptor-operated channels (ROCs); sarcoplasmic reticulum (SR); storage-operated Ca^2+^ entry (SOCE); store-operated channels (SOCs); transient receptor potential channels (TRPs); two pore K^+^ channels (K_2P_); voltage-gated Ca^2+^ channels (VGCC); voltage-gated K^+^ channels (K_v_).

Thus, in the present review, we describe and categorize different expression, functions, regulation, and remodeling of endothelial ion channels (Figure 1) (K^+^, Ca^2+^, Na^+^, and Cl^−^ channels) in PAH. We focus on the potential pathogenic role of ion channel deregulation in the onset and progression of endothelial dysfunction during the development of PAH and its potential therapeutic role.

## 2. Endothelial Cell Dysfunction in PAH

**Figure 2:**
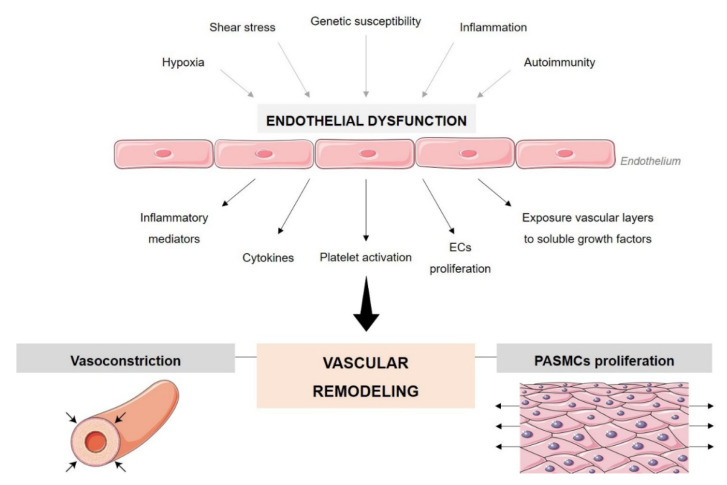
Schematic representation of the role of endothelial dysfunction in pulmonary hypertension and pulmonary vascular remodeling. Endothelial cells (ECs); pulmonary arterial smooth muscle cells (PASMCs).

Endothelial dysfunction is one of the main hallmarks of PAH (Figure 2). Excessive apoptosis of ECs promotes the overgrowth of cells resistant to apoptosis, resulting in uncontrolled angiogenesis that, associated with the disordered proliferation and migration of ECs, results in endothelial accumulation within blood vessels forming plexiform lesions [7]. In addition to ECs, matrix proteins, fibrin, thrombi, platelets, necrotic and fibrotic tissue, and inflammatory cells, are commonly present in plexiform lesions [8]. The stimulus and/or injury that can promote abnormal endothelial proliferation is not known. It may include hypoxia, shear stress, inflammation, and worse response to drugs or toxins in the context of genetic susceptibility [9,10]. ECs can respond to injury in different ways, which can affect vascular remodeling, altering cell proliferation and apoptosis, as well as having functional consequences that can result in a multifaceted imbalance in the production and release of vasoconstrictors/vasodilators, activating/inhibitory growth factors, prothrombotic/antithrombotic mediators, and pro-inflammatory/anti-inflammatory signals [9,11]. In this way, the permeability of the endothelial barrier is compromised and exposure of the vascular layers underlying the endothelium to agents/mediators circulating in the serum increases. Changes in endothelial permeability may occur due to several factors, namely, direct injury, overexpression of vascular endothelial growth factor (VEGF), and activation of inflammatory mediators, cytokines, and oxidants. Mechanical stress also causes morphological changes in ECs, jeopardizing the integrity of the endothelial barrier. The loss of integrity of the endothelial barrier makes the vascular layers more susceptible to proliferative mediators, platelet activation, and release of growth factors that promote PAH-associated vascular remodeling [11].

## 3. Endothelial Cell Ion Channel Function in Pulmonary Arterial Tone

The pulmonary arterial tone is primarily controlled by the resting membrane potential of pulmonary artery smooth muscle cells (PASMCs) and via pulmonary arterial endothelial functions. Membrane permeability to cations and anions plays a vital role in managing intracellular ion homeostasis, cell volume, and excitability [4]. A passive flux of ions across the plasma membrane along the electrochemical gradient is controlled by the activity of ion channels. They are classified into two major classes: the cation channels, which comprise Na^+^, Ca^2+^, and K^+^ channels, and the anion channels, which contain Cl^−^ and bicarbonate channels [4,5].

**Figure 3:**
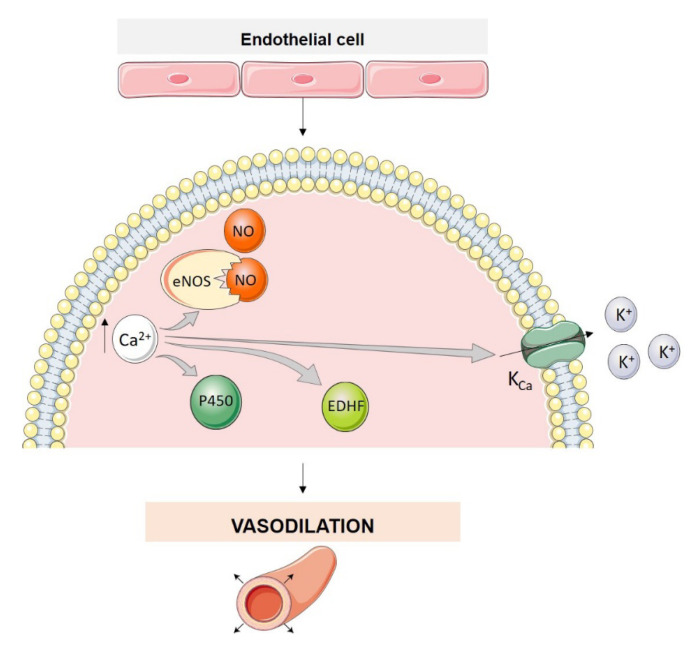
Mechanistic role of Ca^2+^ in endothelial cells of pulmonary artery and its vasodilator and vasoconstrictor effect. Ca^2+^-activated K^+^ channels (K_Ca_); cytochrome P450 (P450); eNOS (endothelial nitric oxide synthase); EDHF (endothelium-derived hyperpolarizing factor); nitric oxide (NO).

Pulmonary vasoconstriction is caused by an increase in cytosolic Ca^2+^ concentration ([Ca^2+^]_cyt_) in PASMCs, while both Ca^2+^-dependent and Ca^2+^-independent mechanisms maintain vasoconstriction. Pulmonary arterial constriction and dilatation, or PASMC contraction and relaxation, are also regulated by factors released from pulmonary artery endothelial cells (PAECs) [4,12]. Activity and expression of the enzymes, for example, endothelial NO synthase (eNOS), required for synthesis and production of these factors (e.g., endothelium-derived relaxing factors, EDRF) depends, at least in part, on variations of [Ca^2+^]_cyt_ in PAECs. Therefore, a rise in [Ca^2+^]_cyt_ in PAECs and PASMCs has dissimilar effects on vascular tone. A rise in [Ca^2+^]_i_ in PAECs causes pulmonary vasodilation by activating different mechanisms including increased activity of eNOS and cytochrome P450, the opening of Ca^2+^-activated K^+^ channels (K_Ca_) which promotes the efflux of K^+^ from PAECs to the intercellular space EC-SMC and endothelium-derived hyperpolarizing factor (EDHF) synthesis, which results in PASMC membrane hyperpolarization (Figure 3) [12,13]. EDHF diffuses into vascular smooth muscle cells (VSMCs) and activates K_Ca_ channels, causing endothelium-dependent hyperpolarization. Furthermore, EDHF can activate transient receptor potential vanilloid channels (TRPV4) with consequent influx of Ca^2+^ into VSMCs [13]. In contrast, increased [Ca^2+^]_cyt_ in PAECs and PASMCs can also induce vasoconstriction and vascular remodeling [12].

Potassium conductance contributes to controlling plasma membrane potential, which regulates [Ca^2+^]. Almost all cells, including PASMCs and PAECs, maintain a negative resting membrane potential (*E*_m_) which is close to the equilibrium potential for K^+^ (*E*_K_). The cell interior is electrically negative with respect to the extracellular compartment; the electrical potential difference ranges from −85 to −60 mV in excitable cells (e.g., PASMCs) and −55 to −30 mV in non-excitable cells (e.g., PAECs) [4,12].

## 4. Classification of Ion Channels in the Pulmonary Circulation and Their Function in PAH Pathophysiology

### 4.1. Ca^2+^ Channels

Cellular Ca^2+^ levels constitute a powerful modulator of cell function. Ca^2+^ levels are differential modulators of several signaling pathways, and several proteins are regulated by Ca^2+^ levels. Therefore, Ca^2+^ levels are involved in different biological functions including the regulation of metabolism, gene transcription, cell proliferation, migration and death, exocytosis and contraction processes, and the release of neurotransmitters [14]. Fluctuations in [Ca^2+^]_i_ are crucial and can even lead to cell death and, therefore, must be highly regulated and controlled to maintain cell homeostasis. [Ca^2+^]_i_ is maintained around 100 nM while the extracellular [Ca^2+^] is between 1–2 mM.

**Figure 4:**
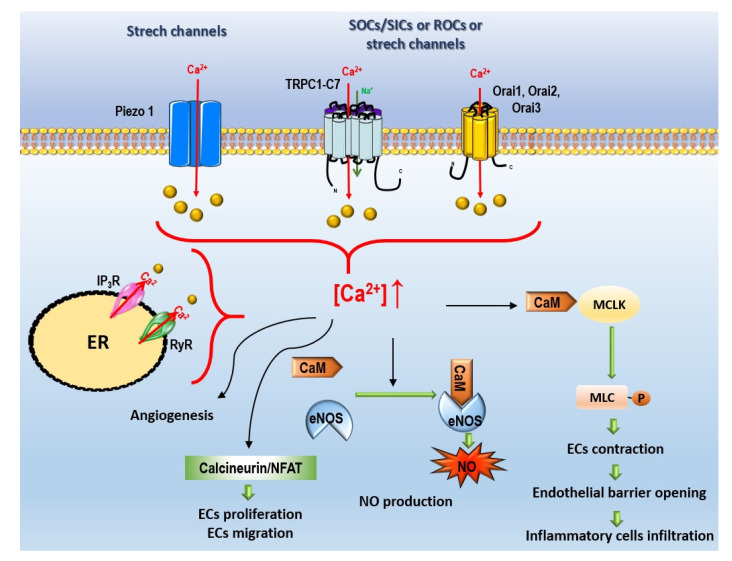
Signaling pathways arising following non-voltage Ca^2+^ activation in PAECs. Calmodulin (caM); endoplasmic reticulum (ER); endothelial cells (ECs); endothelial nitric oxide synthase (eNOS); inositol triphosphate receptor (IP3R); myosin light-chain kinase (MLCK); myeloid cell leukemia (MCL); nitric oxide (NO); nuclear factor of activated T cells (NFAT); store-inhibited channels (SICs); storage-operated Ca^2+^ entry (SOCE); store-operated channels (SOCs); receptor-operated channels (ROC); ryanodin receptor (RyR); transient receptor potential canonical channels (TRPC).

In the pulmonary circulation, regulating Ca^2+^ signaling pathways in ECs and SMCs is highly important for controlling pulmonary vascular tone. Vasoconstriction and vascular remodeling are mediated by the contraction and proliferation of PASMCs, in which Ca^2+^ levels have a major influence. Increase in cytosolic levels of Ca^2+^ is a trigger for vasoconstriction and the proliferation of PASMCs [14]. In PASMCs, Ca^2+^ levels are regulated by two distinct pathways, a voltage-dependent pathway, and a voltage-independent pathway. In this way, the influx of Ca^2+^ can be mediated by three different channels:

(i) Voltage-gated Ca^2+^ channels (VGCC);
(ii) Non-voltage-dependent Ca^2+^ channels (Figure 4):
  (a) Store-operated Ca^2+^ entry (SOCE);
  (b) Non-voltage-dependent store-independent Ca^2+^ entry (SICE), also called receptor-operated Ca^2+^ entry (ROCE);
  (c) Ca^2+^ stretch channels.

#### 4.1.1. Voltage-Gated Ca^2+^ Channels

VGCC, also known as voltage-dependent Ca^2+^ channels, are multimeric proteins composed of four or five subunits. The α1-subunit is the largest and the main subunit responsible for pore formation, pore sensitivity, and the channel’s electrophysiological diversity; it is organized into four homologous domains with six transmembrane segments (S1–S6) in each. Segments S1–S4 work as a voltage sensor, and segments S5, S6, and the P loop form the pore module. These channels are further formed by a hydrophilic protein that forms the ß-subunit, the α2δ-subunit complex, and the γ−subunit [15]. VGCC can be classified into six subtypes—L, T, N, P/Q, and R—according to their functional, electrophysiological, and pharmacological characteristics [16] and are divided into three families. The Cav1 subfamily includes the L-type high voltage-activated (HVA) channels. This subfamily contains the Cav1.1, Cav1.2, Cav1.3, and Cav1.4 channels encoded by the *CACNA1S*, *CACNA1C*, *CACNA1D* and *CACNA1F* genes, respectively. The Cav2 subfamily includes three different types of HVA channels: N-type (Cav2.1), P/Q-type (Cav2.2) and R-type (Cav2.3), which are encoded by the *CACNA1B*, *CACNA1A*, and *CACNA1E* genes, respectively. The Cav3 subfamily includes the T-type low voltage-activated (LVA) channels, which include the Cav3.1, Cav3.2 and Cav3.3 channels, encoded by the *CACNA1G*, *CACNA1H* and *CACANA1I* genes, respectively [15,17]. All channels are activated by membrane depolarization, however, LVA channels are activated slightly above the resting membrane potential, at −60 mV. Furthermore, these channels have a low amplitude channel conductance and a transient decay. On the other hand, the HVA channels require a greater depolarization of the membrane to be activated (−40 mV), have a greater amplitude in the channel conductance and are long-lasting [17].

L- and T-type channels are found primarily in excitable cells, including muscle, neuronal and endocrine cells, inducing Ca^2+^-dependent responses, such as contraction, secretion, and release of hormones [18]. In VSMCs, T-, L-, and P/Q-type channels are expressed [14]. However, the latter has not been studied in the pulmonary vasculature [14]. L-type channels are the first to respond to depolarization in VSMCs, opening the channel and increasing intracellular Ca^2+^ levels, which promotes cellular contraction and vasoconstriction. T-type channels seem to be essentially involved in cell proliferation. Furthermore, T-type channels are associated with the proliferation of cancer cells [19].

The Cav3.1 T-type voltage-gated Ca^2+^ channel is expressed in pulmonary microvascular ECs [20]. Moreover, L- and R-type voltage-gated Ca^2+^ channels were found in other types of ECs [21].

In pulmonary hypertension (PH) pathophysiology: Studies carried out in an animal model of hypoxia-induced PH showed that the regulation of VGCC is essential since the activation of these channels increased pulmonary vasoconstriction in these animals; on the other hand, blockade of the L- and T-type channels attenuated pulmonary artery contraction in these animals. Furthermore, chronic hypoxia (CH) promoted increased Cav1.2, Cav3.1, and Cav3.2 channels compared to control animals [14]. Other studies in animals with CH-induced PH showed that vasoconstriction and proliferation of PASMCs are associated with an increase in the expression of L-type channels, with a consequent increase in intracellular Ca^2+^ levels. Blocking L-type channels has been shown to attenuate hypoxic pulmonary vasoconstriction significantly [14]. The T-type channels, essentially Cav3.1, appear to be involved in PH development; in an animal model of CH-induced PH, blockage/deletion of this channel prevented the development of PH and reduced pulmonary arterial wall remodeling and right ventricular (RV) hypertrophy [22].

In human PASMCs (hPASMCs), Rodman et al. showed that T-type channels are essentially involved in cell cycle progression [23]. These results agree with other studies that showed expression of the T-type Cav3.1 and Cav3.2 channels in the lung and PASMCs of patients with iPAH. The use of TTA-A2 as a T-channel blocker proved to be advantageous, as it prevented cell cycle progression and the growth of PASMCs. In iPAH cells, the signaling carried out by the T-type channels is impaired by the activation of protein phosphatase 2A (PP2A), promoting the activation of ERK1 and p38 and redirecting the activation of Akt1. This failure in T-type channels promotes an increase in the proliferation, survival, and resistance to apoptosis of PASMCs, as evidenced by the phenotype of PASMCs in PAH. Thus, the signaling pathway of T-type channels and PP2A may be an attractive therapeutic target for PAH [24].

Although VGCC elicits a response primarily in excitable cells, the expression of these channels has also been characterized in the brain and adrenal capillary endothelium [25,26] and human coronary artery ECs [27]. Wu et al. demonstrated that pulmonary microvascular ECs express Cav3.1, whereas, in pulmonary macrovascular ECs, such as the pulmonary artery, there is no expression of Cav3.1. However, Cav3.1 channels contribute to the retention of sickle erythrocytes [28]. T-type channels are also expressed in pulmonary capillary ECs and are involved in inflammation processes. A study in Cav3.1^−/−^ and WT mice demonstrated that Cav3.1 channels were involved in acetylcholine (ACh)-mediated endothelium-dependent relaxation of mouse intrapulmonary arteries (IPA), either under physiological or pathological conditions, such as when subjected to CH. At the EC level, ACh induces the influx of Ca^2+^ from Cav3.1, which, consequently, activates the synthesis of NO, leading to NO release, which causes IPA relaxation. ACh-mediated Ca^2+^ entry was impaired in cells from Cav3.1 silenced animals or with the use of T-type channel inhibitors, demonstrating that Cav3.1 channels are involved in the influx of Ca^2+^ and, consequently, in the relaxation of the IPA, which was also less significant in these animals compared to the WT. In addition to Cav3.1 channels, Cav3.2 are also expressed in ECs [29]. T-type Ca^2+^ channel blockade, or the knockout of Cav3.1, reduced pulmonary microvascular ECs proliferation, migration cell-matrix interaction, and in vitro angiogenesis [20]. Unfortunately, to date, little is known about the presence of these channels in PAECs and their involvement in the pathophysiology of PAH.

#### 4.1.2. Non-Voltage-Dependent Ca^2+^ Channels

##### Store-Operated Ca^2+^ Entry 

SOCE is an important mechanism for refilling Ca^2+^ stores. Initially, it was believed to only occur in non-excitable cells. However, evidence shows that it also occurs in excitable cells [30]. SOCE is responsible for refilling internal Ca^2+^ stores in the endoplasmic/sarcoplasmic reticulum (ER/SR) after Ca^2+^ depletion. Upon activation of G-protein-coupled receptors or receptor tyrosine kinase, phospholipase C (PLC) activation converts plasma membrane phospholipids to inositol triphosphate (IP3) and diacylglycerol (DAG). IP3 binds to its receptor (IP3R) and promotes the passive influx of Ca^2+^ from the ER/SR to the cytosol [31]. Store-operated channels (SOCs) have specific characteristics to maintain physical and functional connections between ER and plasma membrane, comprising two major components: the stromal interacting molecules (STIM) and the Ca^2+^ channel Orai [32]. Mammalian cells express two homologs, STIM1 and STIM2. These proteins are transmembrane proteins mostly located in the ER membrane, with a putative Ca^2+^ binding domain in the lumen; they function as Ca^2+^ sensors that monitor the loading level of intracellular Ca^2+^ stores [33,34]. The Orai family can express itself in three distinct isoforms, Orai1, Orai2, and Orai3, and function as a channel, allowing the entry of Ca^2+^. Each Orai monomer comprises four transmembrane domains (TM1-TM4) and contains CAD binding domains at the cytosolic NH_2_ end and at the COOH terminus. Orai proteins are organized in hexamers at the plasma membrane. The maintenance of Ca^2+^ levels by the influx of Ca^2+^ occurs by SOCs, and its activation is essentially dependent on the activity of STIM1. STIM1 is kept inactive throughout the ER and is only activated when Ca^2+^ is released from the ER, concentrating on the ER-plasma membrane junctions, where it interacts with Orai proteins, undergoing conformational changes, oligomerization, binding, and activating Orai proteins [32]. There is also a family of ion channels in several cell types, the transient receptor potential (TRP) family that participates in SOCE [4]. In mammal cells, TRP channels are composed of 28 trp-related genes and divided into seven subfamilies: TRPC (canonical), TRPM (melastatin), TRPV (vanilloid), TRPP (polycystin), TRPA (ankyrin), TRPML (mucolipin), and TRPN (“nompC”, in the potential mechanoreceptor C). They are constituted by six transmembrane domains (TM1-TM6), the pore region is formed between TM5 and TM6, the intracellular N-terminus, and the C-terminus. The N- and C-terminus are protein binding sites that mediate channel trafficking, anchoring, localization, gating, and functional regulation [35]. TRP channels are Ca^2+^-permeable cationic channels, except for TRPM4 and TRPM5 which are only permeable to monovalent ion channels [36]. Thus, TRP channels contribute to the homeostasis of Ca^2+^ levels, either through the influx of Ca^2+^ through plasma membrane or by modulating the resting membrane potential, which controls the entry of Ca^2+^ [34].

TRPC channels have seven known isoforms (TRPC1–7), and we have recently described their contribution to the PAH PASMC phenotype [34]. ECs express many Ca^2+^ channels, including SOCE, SICE, and various Ca^2+^-permeable non-selective cation channels [37]. In murine pulmonary ECs, STIM1 is essential and sufficient for complete SOCE activation associated with TRPC1/C4 channels. In these cells, Ca^2+^ entry mediated by TRPC1/C4 functions is an essential regulator of endothelial barrier integrity [38].

In ECs, there are several expressed TRP channels: from the TRPC family, all seven members seem to be expressed; TRPV1, TRPV2, and TRPV4; of the TRPM family are all expressed in ECs with the exception of TRPM5; and TRPP1 and TRPP2 are also expressed [36]. However, ECs are derived from different vascular beds, and therefore different animal species may express different TRP channels. Bovine aortic ECs express TRPC1, TRPC3, TRPC4, TRPC5, and TRPC6 [39], however, in bovine pulmonary ECs, TRPC3 does not appear to be expressed [40]. Furthermore, TRPC4 and TRPC6 have not been identified in human mesenteric artery ECs [41]. In human coronary artery ECs, some TRPs were identified, the majority of which were affected by shear [42]. TRPV4 is activated by several environmental factors, including by flow, and its expression increased with shear; TRPM7, when subjected to changes in flow, is transferred to plasma membrane and its expression is increased by shear [27].

##### Store-Independent Ca^2+^ Entry, Also Called Receptor-Operated Ca^2+^ Entry

ROCE is a mechanism activated directly in response to extracellular signals and, unlike SOCE, is independent of Ca^2+^ stock or Ca^2+^ release from intracellular deposits [43]. Activation of receptor-operated channels (ROCs) occurs upon binding extracellular ligands to their membrane receptors, including vasoconstrictors and growth factors, such as endothelin-1, serotonin, phenylephrine, histamine, and platelet-derived growth factor [44]. The binding of these ligands contributes to an increased [Ca^2+^]_cyt_ in cells. As in other cell types, increase in [Ca^2+^]_i_ in PAECs could mediate the calcineurin/nuclear factor of activated T cells (NFAT) pathway, which could regulate PAECs proliferation and migration [45].

Despite the same proteins being expressed in SOCE and SICE or ROCE, their activation and regulation mechanisms are distinct. TRPC channels can be activated by the PLC pathway, including DAG [46] and protein kinase C (PKC) or Src kinases [47] to mediate ROCE. Arachidonic acid and its metabolites are suggested to activate the TRPC6 channel [48].

TRPM2 and TRPC6 were found to mediate H_2_O_2_-induced and endotoxin-induced hPAECs hyperpermeability, respectively [49,50]. TRPM2 is involved in several physiological and pathological pathways associated with oxidative stress, and its activation is associated with an increase in [Ca^2+^]_i_. Furthermore, the expression of TRPV1, TRPV2 and TRPM2 channels is also associated with a hypoxic environment, characterized by an increase in oxidative stress [51]. Willette et al. observed that pharmacological activation of TRPV4 retraction/condensation in cultured ECs suggests that it is implicated in microvascular endothelial permeability in the lung, intestine, and kidney [52,53].

In PH pathophysiology: In hPAECs, TRPC1 is expressed [54,55], and TNF-α-induced increase in TRPC1 expression results in endothelial barrier dysfunction, suggesting that the level of TRPC1 expression in ECs is a critical determinant of Ca^2+^ influx and a marker of increased endothelial permeability [55].

Several studies have demonstrated that the TRPC4 channel impacts the development of PAH. Alzoubi et al., after PAH induction in WT and TRPC4^−/−^ rats, found that both groups had similar hemodynamic parameters. However, from histological studies, it should be noted that TRPC4^−/−^ rats had fewer plexiform lesions and fewer obstructed small pulmonary arteries. Thus, it can be inferred that TRPC4 inactivation benefits survival in severe PAH, either by maintaining cardiac output or decreasing occlusive remodeling [56]. Furthermore, TRPC4 appears to be associated with endothelial permeability in PAH; TRPC4 has been shown to increase the frequency of endothelial Ca^2+^ transients in severe PAH, functioning as a source of Ca^2+^ associated with endothelial dysfunction and the pathophysiology of PAH [57]. For instance, ACh recruits TRPC4 to induce NO release and vasodilation in mouse aortic ECs, whereas it impinges on TRPV4 to trigger NO- and EDHF-dependent vasorelaxation in mouse small mesenteric arteries [53].

In PAH PASMCs, TRPC channel dysregulation may be partially responsible for increased Ca^2+^ entry, PASMCs proliferation, and medial vascular pulmonary hypertrophy [34].

Moreover, TRPC1, TRPC3, and TRPC4 are highly expressed in PAECs [58]. Fantozzi et al. suggest that in hPAECs, exposure to CH induces increased expression (mRNA and protein) of TRPC4, leading to an increase in cytosolic Ca^2+^ levels via La^3+^-sensitive TRPC4-encoded SOCs which, in turn, induce an increase in the binding activity of activating protein-1 (AP-1) and consequently promote transcription of AP-1-responsive genes, namely endothelin-1, VEGF, and platelet-derived growth factor. The increased synthesis of these vasoactive and mitogenic factors may influence pulmonary vascular cell proliferation and remodeling. Hence, TRPC4 may be involved in the development of hypoxia-induced pulmonary vascular remodeling in hPAECs [59]. These vasoactive and mitogenic factors, by paracrine mechanisms, could induce the proliferation of PASMCs and, consequently, provoke pulmonary vascular remodeling in patients with PH mediated by hypoxia [60].

Studies carried out by Freichel et al. demonstrated that in TRPC4^−/−^ mice, there was an absence of SOCE in aortic ECs, and ACh-induced endothelium-dependent vasorelaxation was decreased compared to WT animals [61]. Moreover, it was found that additional TRPs were expressed in mice aortic ECs, namely TRPC3 and TRPC6. However, the lack of activity by SOCE in knock-out animals demonstrated that other TRPs do not contribute to SOCE in the absence of TRPC4, highlighting the importance of TRPC4 for the SOCE mechanism in mice aortic ECs, thus demonstrating it to be an essential constituent for channel activation and/or channel pore formation in SOCE [61]. Tiruppathi et al. also demonstrated in TRPC4^−/−^ mice that TRPC4 expression in lung ECs was associated with regulation of vascular tone and endothelial permeability in response to thrombin [62].

Dietrich et al. demonstrated in TRPC6^−/−^ mice that TRPC6 deletion promoted significant increase in TRPC3 expression in some tissues, suggesting that this may be a compensatory mechanism for the action of ROCE [63]. Therefore, TRPC6 has an important role in regulating PASMCs and in the regulation of vascular tone, either by ROCE or SOCE mechanisms.

The TRP channel family plays a crucial role in regulating EC membrane potential and Ca^2+^ homeostasis. Although they are closely related to SOCE and ROCE, the activation of TRP channels may be associated with more direct mechanisms [36]. However, there is still much to understand about their activation and functional relevance in PAH.

In patients with iPAH, SOCE is significantly increased in PASMCs compared to healthy controls [64]. It was found that there was an increased expression of STIM2 and Orai2 in PASMCs from iPAH patients and animals with PH. Upregulation of STIM2 and Orai2 is intrinsically involved in the phenotypic transition of PASMCs from a contractile to a proliferative state; SOCE, STIM2, and Orai2, specifically, play an important role in the proliferation of PASMCs [65]. In transcriptomic analysis, the *ORAI2* gene was also found to be upregulated in the ECs of patients with PAH [66].

In murine pulmonary ECs, Orai1 knockdown had no impact on SOCE [38]. Moreover, in human umbilical vein ECs (HUVECs), the knockdown of TRPC1, TRPC3, and TRPC4, and STIM1 impaired in vitro angiogenesis, whereas the knockdown of Orai1 strongly reduced SOCE but without any effect on HUVECs in vitro angiogenesis [67]. Unfortunately, the role of SOCs in control- and PAH-PAECs angiogenesis is unknown.

In PAH, the pulmonary endothelial barrier is disrupted [2]. Ca^2+^ entry mediated by TRP channels activation can alter endothelial barrier integrity via activation of the calmodulin (CaM)/ myosin light chain kinase (MLCK) pathway, inducing ECs contractions which could facilitate inflammatory cell infiltration and lung oedema [68]. In this way, many TRP channels, including TRPC1, TRPC3, TRPC4, TRPC6, TRPV4, TRPM2, and TRPM4, could regulate systemic endothelial permeability [53]. In mouse lung ECs, Tiruppathi et al. demonstrated that TRPC1 and TRPC4 contributed to thrombin-mediated endothelial permeability [62,69].

Unfortunately, so far, little is known about the SOCE, ROCE, or SICE mechanisms in PAECs and their influence on PAH pathogenesis.

##### Ca^2+^ Stretch Channels

Piezo1 and Piezo2 channels are essential components of stretch-activated ion channels (SACs) [70]. In addition to Piezo channels, some TRP channels, including TRPC1, -C3, -C4, -C5, -C6, TRPV1, -V2, -V4, TRPM4, -M7, and TRPA1 are suggested to be mechanosensitive channels, as recently described by Barbeau et al. [71].

Piezo channels are permeable to cations, including Na^+^, K^+^, Ca^2+^, and Mg^2+^, with a more pronounced selectivity for Ca^2+^. Piezo1 and Piezo2 are described in lung and human isolated pulmonary arteries [4,71]. Piezo1 is expressed in the endothelium, and its knockout profoundly alters vascular architecture [72,73]. Due to its mechanosensitivity, Piezo 1 is involved in blood pressure control [74]. Piezo1 is also expressed in arterial SMCs, contributing to the arterial remodeling occurring in hypertension [75].

In PH pathophysiology: In PAH, Piezo 1 and 2 mRNA expression was unchanged between control and iPAH isolated pulmonary arteries [4]. A recent study showed that in lung and isolated pulmonary arteries from monocrotaline (MCT)-PH rats, Piezo1 protein expression strongly increased compared with control rats. The authors also found increased expression of Piezo1 in lungs from Sugen/hypoxia (SuHx) rats [76]. In hPAECs exposed to hypoxia, they found an increase in Piezo1 expression compared with normoxia-hPAECs and iPAH-hPAECs. The authors found that non-specific blockade of Piezo1 channels with GsMTx4 toxin partially reduced CH PH phenotypes [76]. In contrast, Lhomme et al. demonstrated that endothelial Piezo1 contributed to pulmonary vascular relaxation by controlling endothelial [Ca^2+^]_i_ and, therefore, NO production. They also found that mice with a deletion of the *Piezo1* gene at the endothelial level were not protected against PH induced by CH exposure [77]. Finally, Liao et al. demonstrated upregulation of Piezo1 in iPAH-hPASMCs, contributing to increased [Ca^2+^]_i_ and aberrant iPAH-hPASMCs proliferation [78]. The role of Piezo2 in PAECs is currently unknown. Additional experiments are needed to determine the exact contribution of Piezo channels in endothelial dysfunction occurring in PAH.

### 4.2. Na^+^ Channels

#### 4.2.1. Epithelial Na^+^ Channels

Epithelial Na^+^ channels (ENaC) were first identified in the epithelium of the distal nephron, colon, and lung. They are expressed in the apical plasma membrane by various epithelial tissues of the body, including major cells in the distal nephron of the kidney, bladder epithelial cells, pulmonary airways, distal colon, and salivary glands and ducts (e.g., when sweating) [79]. ENaC comprises two transmembrane domains and contains a large extracellular region abundant in cysteine domains and glycosylation sites [80]. ENaC is a heteromeric protein complex that can be composed of several subunits. However, it is believed that to present its maximum activity, three distinct but similar subunits need to be present [81]. Thus, it is composed of α, β, and γ subunits, which share approximately 30% homology at the amino acid level [80,82]. However, it may include different subunits. The α and δ subunits are fundamental for the formation of the ion permeating pore. In contrast, the β and γ subunits are responsible for maximal channel activity and regulatory function [80,82], the subunits interacting with ENaC regulators, namely, phosphatidylinositol 4,5-bisphosphate (PIP2), phosphoinositide 3 kinase (PI3K), G-protein, and Cl^−^ ions [80].

ENaC is responsible for salt reabsorption, playing a crucial role in controlling the total body homeostasis of salt and water and, consequently, blood pressure [79]. Furthermore, ENaC can respond to mechanical stress, translating it electrically into membrane depolarization [80]. ENaC subunits are also expressed in arterial ECs, so that the vascular mechanosensor can function [82]. In the pulmonary artery, the α-subunit is expressed [80]. Furthermore, studies have shown that the administration of antagonists of these channels, namely amiloride, and benzamil, induces a blockade of myogenic constriction of blood vessels, suggesting that ENaC may be part of a complex of mechanosensitive ion channels necessary for myogenic vasoconstriction [83]. Therefore, ENaC appears to be involved in the regulation of vascular tone. However, although ENaC is expressed in VSMCs and is involved in the myogenic response [84], the expression of ENaC is so far not known in PASMCs [4]. ENaC also appears to be involved in the renin-angiotensin-aldosterone system (RAAS), functioning as a final effector of the RAAS, and is important for Na^+^ balance, controlling renal Na^+^ excretion [85]. In the systemic endothelium, ENaC activation leads to a reduced NO release and consequently vasoconstriction [86]. Nevertheless, the coupling of endothelial mechanics to eNOS function could be a normal physiological mechanism for maintaining appropriate vascular function.

**Figure 5:**
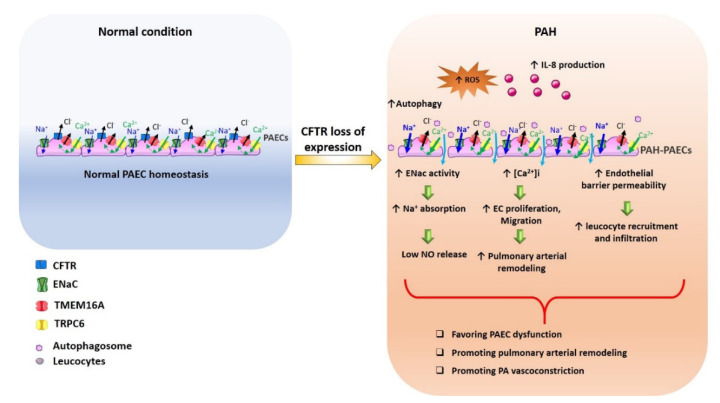
Proposed signaling events arising from CFTR dysfunction in PAECs in PAH. Cystic fibrosis transmembrane conductance regulator (CFTR); epithelial Na^+^ channels (ENaC); nitric oxide (NO); pulmonary arterial hypertension (PAH); pulmonary artery (PA); pulmonary artery endothelial cell (PAEC); reactive oxygen species (ROS).

In PH pathophysiology: In patients with iPAH, the systemic and pulmonary activity of RAAS is increased and is associated with an increase in pulmonary vascular remodeling [87]. Since the activation of RAAS increases the activity of ENaC [88], it is possible that in patients with iPAH, there is also dysregulation of the ENaC. ENaC is functionally coupled with cystic fibrosis transmembrane conductance regulator (CFTR) [89,90]; in cystic fibrosis (CF), the absence of CFTR at the plasma membrane leads to an overactivation of ENaC and consequently excessive Na^+^/fluid absorption contributing to airway surface dehydration and impaired mucociliary clearance of CF airways. We recently demonstrated that CFTR expression is strongly reduced in PASMCs and PAECs from patients suffering from PAH and in experimental models of PH [91]. We hypothesized that the reduced expression of CFTR in PAH-hPAECs could be associated with an increase in ENaC activity (as in CF disease), leading to low NO release and pulmonary artery vasoconstriction (Figure 5). Additionally, CFTR loss of expression/function promotes ECs dysfunction, suggesting the loss of CFTR expression in PAECs from PAH [92]. We imagine that reduced CFTR expression in PAH-PAECs could enhance ENaC function, representing another therapeutic target, as proposed in CF disease [93]. However, there are few studies focusing on the expression of these channels in PAECs and their involvement in the pathophysiology of PAH.

#### 4.2.2. Voltage-Gated Na^+^ Channels

Voltage-gated Na^+^ channels (or voltage-dependent Na^+^ channels) are transmembrane proteins responsible for regulating cell excitability, generating Na^+^ currents that initiate and propagate the action potential, essential in neurons, skeletal muscle, and cardiac myocytes [94,95]. So far, only two Na^+^ channels are known, Nav1 and Nav2. The Nav1 family is divided into nine subtypes, Nav1-Nav9. In mammalian cells, these channels are composed of a large pseudotetrameric pore-forming α-subunit that associates with one or two β-subunits [95]. In skeletal and cardiac muscle cells, action potentials trigger muscle contraction. However, studies demonstrate that vascular smooth muscle action potentials are not dependent on Na^+^ channels, suggesting the absence of Nav channels in these tissues [96]. Some of the eleven genes encoding the α-subunit, *SCN-A*, have been identified in hPASMCs, namely, *SCN2A*, *−3A*, *−4A*, *−8A*, and *SCN1B* and *−2B* for the β-subunit [94]. In PAECs the expression and function of voltage-dependent Na^+^ channels have not yet been investigated, so further studies in this regard should be undertaken.

In PH pathophysiology: Studies have shown that *SCN1B*, which encodes the β1 subunit of the voltage-gated Na^+^ channel, is increased in lung tissues of patients with iPAH [97], suggesting that alterations in the regulation of these channels may be related to the development of pulmonary vasoconstriction [94].

#### 4.2.3. Sodium-Calcium Exchanger

The Na^+^/Ca^2+^ exchanger (NCX) is an important regulator of intracellular Ca^2+^ levels, present in different cells and one of the main factors responsible for cytosolic Ca^2+^ efflux. NCX can carry one Ca^2+^ ion for every three Na^+^ ions entering cells, called the forward operation mode. However, the reverse can also occur, i.e., input of Ca^2+^ through the NCX and output of 3 Na^+^ ions, which is called the reverse operation mode [98,99]. It is believed that in the basal state of animals, the predominant mode is the efflux of Ca^2+^ [100]. The NCX family is encoded by three distinct genes, forming the three isoforms NCX1, NCX2, NCX3. NCX1 is the most abundant isoform expressed in the heart, blood vessel, kidney, brain, and other tissues, whereas NCX2 and NCX3 are predominantly expressed in the brain and skeletal muscle [98]. NCX1 comprises nine transmembrane segments and the large central cytoplasmatic loop. The α-repeat regions are responsible for ion transportation, while the exchanger inhibitory peptide (XIP) region is responsible for regulating Ca^2+^ binding sites; finally, phosphorylation sites are responsible for regulatory properties [99]. NCX is regulated by several physiological factors, namely, the levels of Ca^2+^, Na^+^, and intracellular PIP2 [100].

Of the three NCX isoforms, ECs express only NCX1, which plays a fundamental role in controlling [Ca^2+^]_i_ at rest. Furthermore, it is involved in the Ca^2+^-mediated response activated by endothelium-dependent vasodilators, such as ACh or bradykinin [101]. NCX plays an important role in Ca^2+^-initiated vasodilation in the endothelium of mesenteric resistance arteries. The NCX reverse operation mode activation promotes Ca^2+^ entry associated with the vasodilator response induced by ACh. Furthermore, NCX Ca^2+^ uptake is associated with NO production and the opening of K_Ca_ channels in ECs. Ca^2+^ signaling generated by NCX reverse operation mode activation appears to preferentially activate eNOS, which goes against the fact that both are in the caveolae [101]. Thus, it is important to emphasize the importance of NCX in the endothelium-dependent control of vasomotor tone.

In human coronary artery ECs, NCX1 expression is decreased when ECs are subjected to shear [27]. Furthermore, studies have demonstrated that in hPAECs, NCX1 is involved in alkalinization-induced vasodilation by activating eNOS. Alkalization generated an increase in Ca^2+^ uptake and eNOS activation by NCX1 regulation. Moreover, inhibition of NCX1 with 3′-hydrochloride, 4′-dichlorobenzamyl, suspended the increase in eNOS activity in alkalosis [102].

In PH pathophysiology: In PAH and in several salt-dependent hypertensive animal models, NCX1 is increased in vascular tissue. Mice studies with NCX1^−/−^ and NCX1-overexpressed suggest that NCX plays a major role in several signaling pathways that activate contraction in response to stretch [100].

Wang et al. demonstrated that NCX is inhibited with hypoxic stimuli, which results in a decrease in Ca^2+^ removal and a consequent increase in cytosolic Ca^2+^ levels. This increase in [Ca^2+^]_cyt_ in PASMCs, caused by hypoxia, potentiates vasoconstriction and inhibits vasodilation in the pulmonary artery rings and lungs [103]. In addition, it is a stimulus for the proliferation and migration of PASMCs. Inhibition of NCX blocks the reverse operation mode, which could be a potential therapeutic target for the treatment of PH. This is because the inhibition of NCX and reduction in extracellular Na^+^ attenuate the increase in cytoplasmic Ca^2+^ through the reverse operation mode of the NCX [104].

In PASMCs of patients with iPAH, resting cytosolic Ca^2+^ levels and Ca^2+^ input were increased, which could be a critical mechanism in the vasoconstriction and pulmonary vascular remodeling associated with these patients. NCX1 was increased in the PASMCs of these patients compared to normal individuals, and NCX1, activating the reverse operation mode, caused an increase in cytosolic Ca^2+^ levels. Therefore, NCX1 and its activation in reverse operation mode seem to be one of the mechanisms responsible for increasing intracellular Ca^2+^ in PASMCs of patients with iPAH [105].

So far, studies in the PAECs have not been carried out and, therefore, there is little knowledge in this regard.

#### 4.2.4. Na^+^-H^+^ Exchanger

Na^+^-H^+^ exchanger (NHE) consists of a family of transmembrane proteins. To date, ten isoforms have been identified (NHE1-NHE10). The isoforms share a similar structure and approximately 25–70% amino acid identity [106]. Each NHE isoform is encoded by a distinct gene of the solute transporter family 9 (SLC9), which is divided into three subgroups: *SLC9A*, which is composed of the NHE isoforms 1–9; *SLC9B*, which includes NHA1 (a Na^+^/H^+^ antiporter) and NHA2 (also known as NHE10); and *SLC9C* which includes sperm-specific proteins [107]. The NHE1 isoform is ubiquitously expressed in the plasma membrane of practically all tissues and is therefore considered the maintenance isoform. Although the NHE2-NHE5 isoforms are also expressed in the plasma membrane, the expression of these isoforms is more limited [106].

Although the complete structure of NHE is not fully known, it is recognized that there is a high degree of homology between the isoforms. The structure of mammalian NHE1 consists of two main domains: the amino acid transmembrane domain, which contains twelve transmembrane segments and is the region responsible for ion transport, and the amino acid highly hydrophilic carboxyl-terminus cytoplasmic domain, which is responsible for regulating the exchanger. The H^+^-sensor region, responsible for the channel’s sensitivity to pH, is not fully known. However, it is suggested that it may be located in the transmembrane region [108]. The c-terminal cytosolic domain contains several binding sites necessary for phosphorylation and interaction with regulatory molecules [107,108]. NHE mediates the exchange of extracellular Na^+^ for intracellular H^+^ (output of H^+^ and inflow of Na^+^) and regulate pH homeostasis, cell volume, and transepithelial Na^+^ uptake.

NHE1 is expressed and is involved in pH regulation in mouse and rat PASMCs [107]. However, the contribution of NHE1 to pH regulation may vary from species to species. There are three other cellular pH regulation mechanisms: Na^+^/HCO_3_^−^ co-transport, Na^+^-dependent Cl^−^/HCO_3_^−^ exchanger, and Na^+^-independent Cl^−^/HCO_3_^−^ exchanger. pH_i_ homeostasis regulates PASMCs function in several ways, including controlling vasomotor tone and cell proliferation and migration [109].

In PH pathophysiology: Exposure to CH also appears to influence NHE activity. Studies in PASMCs of mice exposed to CH have shown that there is an increase in basal pH, as well as an increase in NHE activity. Thus, exposure to CH caused an increase in pH and NHE gene and protein expression; these changes induced pH-dependent proliferation of PASMCs, which may contribute to the development of PH [110]. Quinn, Du, Thompson, et al. demonstrated that exposure to hypoxia in rats treated with DMA and ethylisopropyl amiloride (EIPA), NHE inhibitors, significantly reduced pulmonary vascular remodeling and pulmonary artery pressure compared to hypoxic control rats [111]. Furthermore, in NHE1^−/−^ mice, it was found that exposure to hypoxia in these animals did not promote increase in RV systolic pressure (RVSP), RV hypertrophy, or pulmonary vascular remodeling, compared to NHE1-WT mice also exposed to hypoxia. Therefore, deficiency of the *NHE1* gene prevented the development of hypoxia-induced PH and vascular remodeling in mice [112]. Other animal models of PAH, namely SuHx rats, showed that NHE activity was increased in PASMCs isolated from these animals compared to controls. In hPASMCs cultured from patients with iPAH, NHE activity was increased compared to controls [110]. The silencing of NHE1 by small interfering RNA (siRNA) in hPASMCs significantly inhibited cell cycle proliferation and progression, decreasing hypoxia-induced hypertrophy [113].

In hPAECs, NHE1 is expressed on the basolateral surface. Prolonged exposure to hypoxia decreases NHE1 activity. However, NHE1 mRNA levels are not altered, and protein expression is slightly increased. Therefore, exposure to prolonged hypoxia influences NHE1 activity/function. However, this decrease in activity is not related to changes in *NHE1* gene expression. Cutaia, M. V., et al. suggested that changes in NHE1 activity may be associated with the hypoxia-induced alteration of this cytoskeletal component because NHE1 activity is linked to the F-actin cytoskeleton [114]. Changes in NHE1 activity may have consequences for vascular function.

### 4.3. Cl^−^ Channels

#### 4.3.1. Ca^2+^-Activated Cl^−^ Channels–TMEM16 Family

Ca^2+^-activated Cl^−^ channels (CaCCs) are key mediators in several physiological processes, namely, transepithelial secretion, cardiac and neuronal excitation, sensory transduction, photoreception, smooth muscle contraction, regulation of vascular tone, and fertilization. TMEM16A and TMEMB16B are CaCCs and belong to the TMEM16 family of proteins, also known as anoctamin [115,116,117]. The TMEM16 family is composed of ten members (TMEM16A-H, -J, and -K), each of which has ten transmembrane segments with cytosolic terminals -n and -c organized into dimers; these proteins are expressed in the plasma membrane, except for TMEM16E, -H and -K. Furthermore, TMEM16C, -D, -F, -G, and -J are Ca^2+^-dependent phospholipid scramblases [118].

TMEM16A (or anoctamin 1) channels are encoded by the *ANO-1* gene and generate Ca^2+^-activated Cl^−^ currents [117,119]. These channels are expressed in VSMCs, and increase in their activity causes membrane depolarization that, consequently, induces VGCC activation and promotes vasoconstriction. TMEM16A, in addition to being expressed in VSMCs, is also expressed in ECs [120]. Studies have shown that TMEM16A has a particular influence on the endothelium, independently of vascular smooth muscle and adventitia. Ma et al. developed two animal models of transgenic mice: TMEM16A^−/−^ and TMEM16A-overexpressed targeted to the endothelium, showing a significant decrease in blood pressure and an improvement in endothelial dysfunction in TMEM16A^−/−^ mice. In TMEM16A-overexpressed mice, the opposite effects were observed, namely an increase in endothelial dysfunction, suggesting that alterations in TMEM16A expression may be a new therapeutic strategy for diseases associated with endothelial dysfunction [120].

In PH pathophysiology: In the PAH mouse model, TMEM16A expression in pulmonary arteries has been shown to co-localize with a specific VSMC marker that mediates PASMCs proliferation and pulmonary arteriole remodeling [121,122]. Studies in patients with iPAH confirm that TMEM16A is up-regulated, promoting increase in Cl^−^ currents in the PASMCs and inducing proliferation of PASMCs. Inhibition of TMEM16A induces a decrease in the proliferation of iPAH-PASMCs. On the other hand, overexpression of TMEM16A in PASMCs from healthy patients caused a similar phenotype to iPAH in these patients [123]. Therefore, TMEM16A regulates VSMCs proliferation and remodeling and is involved in the cell cycle [124].

Recent studies indicate that TMEM16A contributed to the pathogenesis of PAH by increasing its activity, which promoted increased Cl^−^ current in the cell membrane of PAECs from patients with iPAH. To understand the consequences of increase in TMEM16A in iPAH-PAECs, mimicking the overexpression of TMEM16A in healthy PAECs resulted in functional consequences of the increased activity of TMEM16A, namely, changes in Ca^2+^ dynamics and eNOS activity, decreasing the production of NO, the proliferation of PAECs, wound healing, tube formation and ACh-mediated relaxation of the pulmonary arteries [125]. Furthermore, TMEM16A is located in the mitochondria of pulmonary ECs, and its activation induces apoptosis through an mtROS–p38–caspase-3 pathway. As TMEM16A is increased in patients with iPAH, it promotes apoptosis in ECs in these patients [126]. Further studies should be performed to understand whether TMEM16A is a viable candidate to reverse the hyperproliferative phenotype associated with PAH by TMEM16A silencing.

#### 4.3.2. Cystic Fibrosis Transmembrane Conductance Regulator

The CFTR is a low-conductance, cyclic nucleotide-regulated Cl^−^ channel. This protein is 180kDa in size and is composed of two domains, the first includes six transmembrane-spanning α-helices, a first nucleotide-binding domain (NBD-1) that binds to ATP, and a large regulatory domain which is rich in cyclic adenosine monophosphate (cAMP)-dependent kinase and PKC phosphorylation sites; the second domain is composed of six transmembrane-spanning α-helices and a second nucleotide-binding domain that binds ATP [127]. CFTR is primarily expressed in epithelial cells, however, it can also be expressed in non-epithelial tissues, such as cardiac muscle cells, ECs, and SMCs [128]. Mutations in the *CFTR* gene are intimately involved in CF [127]. Robert et al. demonstrated that these channels are expressed in pulmonary arteries and are involved in endothelium-independent pulmonary vasorelaxation [128]. In animal models with CFTR deficiency, either by silencing or blocking, there was a decrease in ECs proliferation, migration, and autophagy; however, defective CFTR function led to ECs activation and a persistent pro-inflammatory state of the endothelium with increased leukocyte adhesion [92].

In PH pathophysiology: Studies have demonstrated that mice with defective CFTR in the lungs are moderately protected against PH and pulmonary arterial remodeling, probably due to the CFTR/TRPC6 protein complex [129]. Thus, CFTR is involved in the regulation of hypoxic pulmonary vasoconstriction, and CFTR dysfunction is related to the impairment of endothelial monolayer integrity and eNOS function [130]. Q. Yang et al. argue that CFTR dysfunction may be involved in the development of PH because the decreased expression of CFTR has a protective effect against primary PH [131].

Recent studies have shown that CFTR expression is strongly decreased in PASMCs and PAECs in human and animal models of PH. Furthermore, CFTR activation induces relaxation in human, pig, and rat pulmonary arteries. Long-term inhibition of CFTR in rats causes an increase in RVSP, which is intimately associated with increased proliferation in situ of pulmonary ECs. Therefore, CFTR inhibition promotes increased proliferation and reduces pulmonary artery relaxation [91]. As in CF, the lack of CFTR could also dysregulate ENaC and TMEM16A [132]. Indeed, in airway epithelial cells, CFTR and TMEM16A are both localized at the plasma membrane and could be co-immunoprecipitated. Moreover, CFTR-currents are reduced by over-expression of TMEM16A. Furthermore, CFTR was also functionally and reciprocally coupled with TRPC6. The loss of plasma membrane CFTR localization induced an increase in TRPC6-Ca^2+^ and the CaCCs current [133,134]. We could imagine similar regulation in PAECs. Since CFTR expression is lost in iPAH, taking advantage of the knowledge obtained in CF, we could expect that CFTR loss of expression in iPAH would mediate ENaC, TMEM16A and TRPC6 misregulation (Figure 5). However, the role of CFTR in PAECs isolated from PAH patients is unknown and needs to be investigated to determine the contribution of CFTR in endothelial dysfunction in PAH.

### 4.4. K^+^ Channels

#### 4.4.1. Voltage-Gated K^+^ Channels 

Voltage-gated-K^+^ channels (K_v_) are K^+^ channels composed of an α-subunit formed by six transmembrane domains, four voltage sensor domains, and two domains that include the pore; and a β-subunit composed of an intracellular domain. K_v_ need to be in tetrameric conformation to be functional. They could form homo- or heterotetramers with four α subunits and four β subunits. Activation of K_v_ channels depends on membrane voltage. They are opened by membrane depolarization, leading to efflux of K^+^, resulting in membrane repolarization at the resting membrane potential. Therefore, K_v_ channel functions are to maintain the resting membrane potential on excitable cells, including VSMCs, where they are essential to the regulation of vascular tone and cell viability [135,136]. 

Few studies have studied the K_v_ channel in PAECs. Whole-cell patch-clamp experiments showed the presence of K_v_ current (I_Kv_) in ECs from several origins, including rabbit cerebral ECs [137] and rat cardiac microvascular ECs [138]. Moreover, murine cerebral arteriole ECs expressed K_v_1.2 and K_v_1.3 isoforms [139]. In rat mesenteric ECs, K_v_7.1, K_v_7.4, and K_v_7.5 channels were expressed, controlling the mesenteric arterial tone in an endothelium-dependent manner and interaction with inward rectifier K^+^ channels family 2 (K_ir_2.x) [140].

In PH pathophysiology: In the endothelium of small pulmonary arteries, Hogg et al., showed, using an immunostaining approach, the presence of K_v_1.5 in freshly isolated PAECs. By whole-cell patch-clamp experiments, they also found that 90% of the PAECs had a K^+^ voltage-dependent current corresponding to I_Kv_ and that 10% of remaining PAECs had an electrophysiological signature corresponding to I_Kir_ [141].

More recently, Babicheva et al. demonstrated the expression of K_v_1.5 and K_v_1.2 proteins in hPAECs. In contrast to their expression in hPASMCs isolated from iPAH patients, they showed that K_v_1.5 and K_v_1.2 expression was unchanged in hPAECs isolated from iPAH patients [142].

#### 4.4.2. Ca^2+^-Activated K^+^ Channels

K_Ca_ channels are composed of six transmembrane segments and one pore domain. Two of these segments form the pore of the channel. The K_Ca_ channel family has a very close homology to K_v_ channels. The main difference is their sensitivity for [Ca^2+^]_i_. K_Ca_ are activated by an elevation of [Ca^2+^]_i_, while K_v_ are insensitive to [Ca^2+^]_i_. Therefore, EC membrane hyperpolarization, mediated by K_Ca_ opening, also plays a role in NO release and NOS activation by regulating Ca^2+^ influx [143].

There are three subtypes of K_Ca_ channels: the big conductance K_Ca_ channels (BK_Ca_) which have the biggest single-channel conductance among K^+^ channels (100–300 pS), with activation mediated by both voltage and [Ca^2+^]_i_ [144]; the intermediate-conductance K_Ca_ channels (IK_Ca_) which have a conductance of 25–100 pS and for which the activation is not dependent on voltage, but only on [Ca^2+^]_i_; and the small conductance K_Ca_ channels (SK_Ca_) with a conductance of 2–25 pS. In contrast to other K_Ca_, SK_Ca_ activation is voltage-independent, and their I/V relationship shows an inward rectification [145].

Several studies have shown that IK_Ca_ and SK_Ca_ are expressed in different EC types, including human mesenteric artery ECs (hlK1 channel) and porcine coronary endothelium (SK_Ca_) [146,147,148]. In the guinea-pig carotid artery, Gluais et al. showed IK_Ca_ and SK_Ca_ endothelium-dependent hyperpolarization through ACh response and participation in EDHF release [149]. Furthermore, the SK_Ca_ channel, SK3, was expressed in rat carotid artery ECs and played a role in the EC hyperpolarization and the generation of EDHF [150]. In HUVECs, SK_Ca_ and IK_Ca_ activation are implicated in the NO production pathway [143].

BK_Ca_ channels (BK_Ca_ α-subunit, BK_Ca_ β1-subunit, BK_Ca_ β2-subunit, BK_Ca_ β4-subunit) are expressed in rat PAECs at mRNA and protein levels. The pharmacological activation of BK_Ca_ with the BK_Ca_ channel opener NS1619 led to PAEC hyperpolarization followed by pulmonary arterial vasodilatation (via the production of NO), which was strongly reduced in ECs denuded pulmonary arteries, confirming the importance of BK_Ca_ in PAECs [151]. Moreover, the role of large-conductance K_Ca_ in the pulmonary circulation was recently revised by Guntur et al. [152].

In PH pathophysiology: In pulmonary vasculature, K_Ca_2.3 and K_Ca_3.1 channels are expressed in PAECs, and K_Ca_2.3 contributes to endothelial-dependent pulmonary arterial relaxation [153]. K_Ca_3.1 protein expression is decreased in pulmonary arteries from CH-PH rats. This dysregulation could be linked to impaired endothelium-dependent pulmonary artery relaxation in CH-PH rats [154].

Babicheva et al. showed the expression of KCNMB1 (coding for BK_Ca_ β1-subunit) in hPAECs. They found that BK_Ca_ β1-subunit protein expression was increased in iPAH-hPAECs compared with control-hPAECs, and that the increased expression was correlated with miR-222 over-expression or miR-138 down-expression in iPAH-hPAECs [142].

Recent results have confirmed the mRNA expression of KCNMB1, KCNMB3-4, and KCNMA1 in hPAECs, while KCNMB2 seems not to be expressed. It was found that BK_Ca_ activation with NS1619 in hPAECs could control cytokine production (CCL-2) induced by lipopolysaccharide (LPS) exposure by reducing proinflammatory and promoting anti-inflammatory signals. The phenomenon occurred independently of [Ca^2+^]_i_ variation but probably via unknown mechanisms dependent on the resting membrane potential [155]. Ex vivo, the NS1619 compound induced endothelium-dependent pulmonary arterial relaxation, dilation and a reduction in pulmonary arterial pressure. Moreover, in vitro NS1619 application induced important PAEC hyperpolarization and increased production of NO [151].

In vivo chronic administration (preventive protocol) of a new selective BK_Ca_ channel opener (extracted from a family of tetrahydroquinolines) significantly reduced the development of PH in MCT-induced PH models [156].

#### 4.4.3. Two Pore Potassium Channels

Two pore potassium channels (K_2P_) are also called the background K^+^ conductance channels and are constituted by six sub-families: the TWIK, the TWIK-related K^+^ channel/TWIK-related arachidonic acid-stimulated K^+^ channels (TREK/TRAAK), the TASK, the TWIK-related alkaline pH-activated K^+^ channels (TALK), the tandem pore domain halothane-inhibited K^+^ channels (THIK) and the TWIK-related spinal cord K^+^ channels (TRESK) [157]. They are composed of four transmembrane domains that make a functional dimer and form the channel’s pore. Several mechanisms regulate them through pH variation, G-proteins, oxygen level, and shear stress, mostly by controlling the resting membrane potential of the cells.

In 2004, immunostaining experiments on rat pulmonary arteries revealed the presence of TASK-1 and TASK-2 on the EC layer but in smaller proportion than in the PASMCs, while TREK-1 and TWIK-2 proteins seemed not to be expressed in PAECs [158].

In PH pathophysiology: In 2013, whole-genome sequencing identified several loss of function mutations in the *KCNK3* gene encoding for TASK-1 in PAH patients [159]. To date, 16 different *KCNK3* mutations have been identified in 23 patients. Importantly, PAH patients with *KCNK3* mutations were younger at diagnosis than iPAH patients and were more severe than iPAH patients, as indicated by a higher mean pulmonary arterial pressure (mPAP) (76 mmHg compared to 56.4 mmHg) [160], suggesting the crucial role played by KCNK3/TASK1 in PAH pathogenesis. *KCNK3* is sensitive to extracellular pH variations (fully inhibited at pH 6.4, 50% activated at physiological pH 7.4, and fully activated at pH 8.4). Several molecules inhibit *KCNK3*, such as 4-aminopyridine and several agonists coupled with Gq protein receptors. We confirmed the critical role of KCNK3/TASK1 dysfunction in the pathogenesis of PAH at PASMCs and RV cardiomyocyte levels by several approaches, including *Kcnk3*-LOF-mutated rats [6,161,162,163].

Regarding the role of KCNK3/TASK1 in PAECs, we also found, by whole-cell patch-clamp recording, that KCNK3/TASK1 was functionally expressed in freshly isolated rat PAECs. Moreover, we demonstrated that I_TASK1_ was severely reduced in PAECs isolated from MCT-PH rats (7 days after MCT exposure), correlated with PAEC membrane depolarization [161].

We recently analyzed the consequences of knockdown *KCNK3* in hPAECs by a siRNA strategy using an unbiased proteomic approach. The proteomic analysis revealed 157 proteins upregulated and 247 downregulated in siKCNK3-hPAECs compared with sicontrol-hPAECs. These dysregulated proteins were linked to dysregulation of several signaling pathways involved in controlling cell proliferation, cell migration, cell apoptosis, and cell metabolism, including the eukaryotic initiation factor (EIF2) pathway, the mTOR signaling pathway, and the superpathway of methionine degradation. These results suggest that KCNK3/TASK1 plays a key role in these hPAECs functions, contributing to PAH pathogenesis at the PAEC level [160]. Using *Kcnk3*-LOF-mutated rats, we showed that KCNK3/TASK1-LOF led to a reduction in eNOS expression, activation of the endothelial-to-mesenchymal transition (endoMT) transcription factor, altered expression of molecules crucial to maintaining endothelial integrity (CD31, vWF), and desensitization of pulmonary arteries to EDHF, favoring pulmonary artery remodeling and pulmonary artery vasoconstriction [162].

#### 4.4.4. ATP-Sensitive K^+^ Channel

ATP-sensitive K^+^ channel (K_ATP_) represents a K^+^ channel family sensitive to intracellular ATP concentration. K_ATP_ is composed of K_ir_ channels, K_ir_6.1 or K_ir_6.2, which correspond to the channel’s pore, and sulfonylurea receptors with regulatory subunits, SUR1, SUR2A, or SUR2B. SUR regulatory subunits are members of ATP-binding cassette (ABC) transporters that use ATP hydrolysis to mediate a large variety of functions.

A tetrameric conformation forms K_ATP_ with four K_ir_ and four SUR subunits. They are known to take part in the regulation of the resting membrane potential.

K_ir_6.1, K_ir_6.2, and SUR2 mRNA are expressed in ECs from different origins, including guinea pig heart, rat aorta, rat brain microvascular, rabbit aorta, and human coronary artery [164,165,166,167].

Endothelial K_ATP_ function is associated with NO production, mediated by adenosine, which could activate the K_ATP_ channel [168]. Endothelial K_ATP_ activation could also directly act on NO synthesis participating in SMC relaxation. Aziz et al. showed, in the aorta and mesenteric ECs from mice, that K_ATP_ was mainly composed of the SUR2B/K_ir_6.1 channel [169]. Moreover, using EC-specific *kir6.1*^−/−^ mice, Li et al. found that endothelial K_ir_6.1 contributed to systemic blood pressure regulation upon pathological challenge (high salt diet) [170], demonstrating the potential protective role of the endothelial K_ATP_ during the development of systemic hypertension.

Regarding pulmonary vascular ECs from rat and bovine tissue, K_ir_6.1 mRNA expression was not detected, while K_ir_6.2 expression (mRNA and protein) was seen and increased under shear stress exposure [171].

In PH pathophysiology: In 2018, 12 heterozygous mutations on the *ABCC8* gene encoding for SUR1 were discovered in PAH patients [172]. *ABCC8* carrier PAH patients were younger at diagnosis (14 years compared to 42 years for iPAH patients) but had similar mPAP. Our preliminary results found expression of SUR1 and K_ir_6.2 on hPAECs from control and PAH patients. We also found that preventive and curative selective SUR1 activation in experimental PH induced by MCT- or CH-exposure improved PH phenotype, confirming the contribution of SUR1/ABCC8 in the development of PH [173].

Li et al. demonstrated that in vivo preventive treatment with iptakalim, a non-selective K_ATP_ activator, improved experimental PH induced by MCT exposure in rats [174]. Another study showed that nicorandil treatment (non-selective K_ATP_ activator) on MCT-PH rats attenuated PH phenotype and enhanced eNOS expression [175].

Together these results suggest that K_ATP_ channels could be an attractive therapeutic target to fight against PAH. However, the need for a more potent selective molecule of K_ATP_ isoform is essential for determining each isoform’s contribution to PAH physiopathology.

#### 4.4.5. Inward Rectifier Channel Family

There are seven K_ir_ families (K_ir_1.x to K_ir_7.x) composed of two transmembrane domains forming the channel’s pore. They display inward rectification of K^+^ influx, and intracellular molecules regulate them, such as Mg^2+^ and polyamines [176].

In bovine primary PAECs, several K_ir_ subtypes were expressed, including K_ir_2.1, K_ir_2.2, K_ir_2.3, K_ir_2.4, K_ir_3.1, K_ir_4.1, and K_ir_5.1. Only the K_ir_1.1 subtype was not detected. In this study, the authors found that K_ir_2.1 expression was regulated by calmodulin-dependent protein kinase II (CaMKII) [177].

Recently, K_ir_2.1 was expressed in mice ECs from mesenteric arteries where its function was essential for NO-dependent vasodilatation and eNOS phosphorylation. Using *kir2.1^+/−^* mice, it was found that K_ir_2.1 played a major role in flow-induced endothelium-dependent vasodilatation [178]. This phenomenon, and the expression of all K_ir_X.X isoforms, remains unknown in hPAECs or their contribution to PAH pathogenesis.

## 5. Emerging Ion Channel Targets for PAH Therapy

### 5.1. Ca^2+^ Channels

#### 5.1.1. Direct Pharmacological Action

The L-type Ca^2+^ channel blockers, nifedipine, diltiazem, and amlodipine, are currently the only therapy used in PAH acting on ion channels [179,180]. The use of these inhibitors cause significant clinical and hemodynamic improvement in patients with PAH. However, this therapy approach is not possible for all patients and, therefore, they should undergo an acute trial with a pulmonary vasodilator, NO, beforehand. Patients with iPAH underwent oral treatment with Ca^2+^ channel blockers (CCBs), but only 54% responded positively. Patients who responded positively to treatment demonstrated clear improvement including less severe disease, a higher proportion of patients in NYHA functional class II, better 6MWD test and less severe hemodynamic parameters, as well as improvement after one year of treatment [180]. Although they are already used in PAH therapy, there are no known advantages in terms of their action at the endothelial level.

#### 5.1.2. Indirect Pharmacological Action

Blocking T-type Ca^2+^ channels has a greater impact on inhibiting cell proliferation than inhibiting L-type Ca^2+^ channels. Mibefradil, a T-type Ca^2+^ channel blocker, completely inhibits cell proliferation and prevents entry into the cell cycle. On the other hand, diltiazem, an L-type Ca^2+^ channel blocker, did not show marked effects. Selective blocking of Cav3.1 expression with siRNA completely inhibited proliferation and prevented entry into the cell cycle [23]. In animal models of CH-induced PH, blockade of T-type Ca^2+^ channels by TTA-A2 prevented induced PH, reduced right cardiac hypertrophy, and induced pulmonary artery remodeling. In addition, TTA-A2 decreased PASMCs proliferation and prevented vascular hyperreactivity [22]. Verapamil and SKF 525A, known antagonists of Ca^2+^ channels, were also responsible for inhibiting hypoxic pulmonary vasoconstriction, suggesting that this was essentially mediated by the transmembrane influx of extracellular Ca^2+^ [181].

There are several pharmacological compounds with a preventive effect on PAH, however, they do not specifically affect ECs. Sildenafil, for example, an inhibitor of phosphodiesterase type 5, has a positive impact on therapy in patients with PAH [182,183,184] as it has an inhibitory effect on the proliferation of hPASMCs. The antiproliferative effect of sildenafil may be related to the downregulation of TRPC1 gene expression [185]. Pyrazol2, a blocker of TRPC channels, has been shown to significantly attenuate RV hypertrophy and PAH in mice with MCT-induced PAH [186]. Furthermore, iloprost, the synthetic analog of prostacyclins, substantially decreased the expression of TRPC3 in iPAH-PASMCs, consequently inducing a decrease in the proliferation of PASMCs; suggesting that the vasodilatory and antiproliferative effects of prostacyclin and its analogs may be involved in the inhibition of TRPC expression in PASMCs [187]. Therefore, targeting PAH therapies to TRPC channels may be a useful therapeutic strategy. At the EC level, further studies should be carried out to understand the interactions of these channels in ECs and, consequently, in PAH.

### 5.2. Na^+^ Channels

The regulation of NHE is essential to maintain intracellular pH, however, it only plays a permissive role in the proliferation of PASMCs, and little is known about its action at the level of PAECs. However, NHE inhibitors appear to be attractive therapeutic targets for PAH.

#### Indirect Pharmacological Action

The use of NHE inhibitors, such as dimethyl amiloride and ethylisopropylamiloride, have been shown to significantly reduce pulmonary vascular remodeling in a hypoxia-induced PH animal model [111]. Sabiporide, a member of the NHE1 inhibitor family, also inhibits the proliferation and migration of hPASMCs, blocking cell progression [188]. Cariporide, another NHE inhibitor, attenuates the development of right heart failure. The use of this inhibitor in mice with MCT-induced PH promoted a decrease in RVSP and RV hypertrophy. In addition, cariporide attenuated necrosis fibrosis and induced RV myocardium mononuclear cell infiltration [189].

Another mechanism to inhibit NHE is the upstream inhibition of hypoxia-inducible factor 1 (HIF-1). HIF-1 is a transcription factor that upregulates the NHE1 gene in response to hypoxia [107]. Studies show that digoxin, a cardiac glycoside, inhibits the transcriptional activity of HIF-1. Digoxin treatment in mice with hypoxia-induced PH has been shown to attenuate increase in RVSP, RV hypertrophy, and pulmonary vascular remodeling and to delay the progression of established PH. Furthermore, digoxin treatment prevented increase in NHE1 expression in PASMCs. Treatment with acriflavine, another HIF inhibitor, but by a different path, also prevented the development of PH [190]. However, to date, at the level of PAECs, little is known about the inhibition of these channels and their possible involvement in the pathophysiology of PAH.

### 5.3. Cl^−^ Channels

TMEM16A expression and function are significantly increased in PAH. Studies on iPAH-PASMCs have shown that TMEM16A is upregulated, increasing the Cl^−^ current. Inhibition of TMEM16A by benzbromarone (BBR) reversed membrane depolarization in iPAH-PASMCs to healthy levels. Chronic treatment with BBR in animal models of PH promoted a considerable reduction in RVSP and pulmonary artery muscularization, demonstrating a potent attenuation of vascular remodeling. BBR has been approved for the treatment of gout in humans, with a maximum oral dose of 200 mg. Therefore, more studies should be undertaken to understand the possible effects of BBR in the treatment of iPAH and to determine recommended doses [123].

Treatment with T16Ainh-A01, an aminophenylthiazole inhibitor of TMEM16A, was shown to be beneficial in MCT-induced PAH mice. Administration of T16Ainh-A01 significantly alleviated pulmonary arteriole remodeling and RV hypertrophy, decreased pulmonary artery pressure, and decreased upregulation of proliferating nuclear antigen (PCNA) in pulmonary arteries. T16Ainh-A01, in addition to inhibiting the activity of the TMEM16A channel, also suppressed its effect on cell proliferation. Administration of T16Ainh-A01 significantly improved PAH. However, it did not lead to full recovery. Thus, T16Ainh-A01 appears to be a promising drug in improving vascular remodeling [121]. Increase in TMEM16A expression in healthy PAECs has functional consequences in PAECs, such as changes in Ca^2+^ dynamics and eNOS activity, decreasing NO production, promoting PAECs proliferation, wound healing, tube formation and relaxation of pulmonary artery mediated by ACh [125]. Studies should be carried out to understand whether silencing TMEM16A can reverse the PAH-induced phenotype in healthy PAECs and whether it will be a therapeutic candidate for PAH.

### 5.4. K^+^ Channels

#### 5.4.1. Direct Pharmacological Action

PAH is associated with a loss of K^+^ channel expression and activity. Pozeg et al. demonstrated that in vivo gene transfer of the Kv1.5 channel in CH-induced PH rats promoted a significant improvement in PH as well as hypoxic pulmonary vasoconstriction [191]. Furthermore, studies have demonstrated that *KCNA5* gene transfer, in addition to increasing K^+^ currents, increased caspase-3 activity and accelerated apoptosis. Thus, the induction of apoptosis through gene therapy can be a fundamental strategy to prevent the progression of pulmonary vascular wall thickening and for treating iPAH patients [192]. However, the authors only focused on *KCNA5* re-expression in PASMCs. We did not find any information on the consequences of *KCNA5* re-expression in PAECs.

Nicorandil, a nicotinamide ester, is a K_ATP_ channel opener with a NO release vasodilator function. In an animal model of MCT-induced PAH, nicorandil protected the pulmonary endothelium from damage, reduced apoptosis, and attenuated PAH development through upregulation of eNOS expression anti-apoptotic factors, mediated by PI3K/Akt and ERK1/2 signaling pathways [175]. In this way, nicorandil could be an attractive therapeutic target at the endothelial level for the treatment of PAH. In addition, iptakalim (2,3-dimethyl-*N*-(1-methylethyl)-2-butanamine hydrochloride), a compound responsible for opening K_ATP_ channels, is involved in inhibiting PASMCs proliferation and pulmonary vascular remodeling by downregulation of PKC-alpha [193]. In several animal models of PH, treatment with iptakalim attenuated induced PH and pulmonary arterial wall remodeling. In addition, it attenuated the inflammatory response and prevented endothelial damage [174,194]. However, at PAEC level, little is known about its therapeutic effect.

#### 5.4.2. Indirect Pharmacological Action

Levosimendan is a calcium-sensitizing medication used clinically to treat right heart failure in PH. Studies in an MCT model of PH demonstrated that levosimendan attenuated increase in pulmonary vascular medial wall thickness and significantly decreased the proliferation of PASMCs in vitro and in vivo. This effect appeared to be K_ATP_-dependent. In addition, levosimendan increased endothelial NO generation and decreased the expression of inflammatory genes in ECs. Thus, levosimendan reduces pulmonary vascular remodeling, probably due to an antiproliferative and anti-inflammatory effect, making it an interesting target for treating PAH [195].

In 2013, six new heterozygous missense variants were identified in *KCNK3*. Electrophysiological studies found that all variants resulted in the loss of function. The administration of the phospholipase A2 inhibitor ONO-RS-082 reactivated the current of some *KCNK3* mutants. In this sense, ONO-RS-082 could be a new therapeutic approach for PAH [159]. Several studies were carried out after these discoveries. Antigny et al. demonstrated that *KCNK3* expression and function were reduced in patients with PAH and rats with MCT-induced PH. In vitro, using the patch-clamp technique in PASMCs and PAECs, it was found that ONO-RS-082 promoted an improvement in the endogenous *KCNK3* current in rats. Furthermore, it was found that, although treatment with ONO-RS-082 was ineffective in reducing PH symptoms, preventive therapy with ONO-RS-082 improved hemodynamically induced PH symptoms in RV hypertrophy and pulmonary vascular remodeling [161]. Thus, since the loss of *KCNK3* may be a key event in the pathogenesis of PAH, there is interest in furthering this therapeutic approach.

#### 5.4.3. Untested Pharmacological Compounds

K_v_ channels are downregulated in patients with PAH. Morecroft et al. studied the influence that flupirtine, a K_v_7 channel activator, had in two models of induced PAH. In both animal models, an increase in mean RV pressure (mRVP) was associated with remodeling of the pulmonary vasculature and RV hypertrophy. In the presence of flupirtine, these effects were attenuated as a specific result of increase in mRVP through the activation of the K_v_7 channel. Thus, therapies that involve the activation of K_v_7 channels appear to be beneficial in treating PAH of different etiologies [196]. In addition, flupirtine reduced vascular resistance in the lung and inhibited hypoxic PH [197].

## 6. Conclusions

Through this review, we have demonstrated the relevance of the expression, activation, regulation, and function of various ion channels (K^+^, Ca^2+^, Na^+^, and Cl^−^ channels) in the pathophysiology of PAH, focusing primarily on the endothelial dysfunction characteristic of the disease. There is no treatment capable of curing PAH, and the most effective therapy is lung transplantation for eligible patients who still have high morbidity. Recent discoveries and identification of the role of some ion channels in PAH have revived interest in studying them as potential therapeutic targets in PAH, essentially at the endothelial level.

This review summarizes the current knowledge on the expression and activation of ion channels in PAECs and their influence on the pathophysiology of PAH, considering studies in humans and several experimental animal models that mimic PAH. Ion channels play a crucial role in the pathophysiology of the disease and can be carefully considered as new therapeutic targets relevant for PAH. However, at the level of PAECs, little knowledge has been acquired to date. Therefore, more studies should be carried out to deepen understanding of the effects of different ion channels on endothelial dysfunction. Indeed, a greater understanding of the role of the endothelium in PH should facilitate the evolution of newer, targeted therapies.

## Data Availability

Not applicable.
